# The "ComPAS Trial" combined treatment model for acute malnutrition: study protocol for the economic evaluation

**DOI:** 10.1186/s13063-018-2594-7

**Published:** 2018-04-24

**Authors:** Natasha Lelijveld, Jeanette Bailey, Amy Mayberry, Lani Trenouth, Dieynaba S. N’Diaye, Hassan Haghparast-Bidgoli, Chloe Puett

**Affiliations:** 1 No Wasted Lives, Action Against Hunger UK, London, UK; 2 Department of Population Health, London School of Hygiene and Tropical Medicine, London, UK; 3 International Rescue Committee, New York, NY USA; 4 Action Against Hunger USA, New York, NY USA; 5 Action Contre la Faim (ACF), Paris, France; 6 Institute for Global Health, University College London, London, UK

**Keywords:** Cost-effectiveness, Severe acute malnutrition, Moderate acute malnutrition, Community management of acute malnutrition, Cost-consequence analysis

## Abstract

**Background:**

Acute malnutrition is currently divided into severe (SAM) and moderate (MAM) based on level of wasting. SAM and MAM currently have separate treatment protocols and products, managed by separate international agencies. For SAM, the dose of treatment is allocated by the child’s weight. A combined and simplified protocol for SAM and MAM, with a standardised dose of ready-to-use therapeutic food (RUTF), is being trialled for non-inferior recovery rates and may be more cost-effective than the current standard protocols for treating SAM and MAM.

**Method:**

This is the protocol for the economic evaluation of the ComPAS trial, a cluster-randomised controlled, non-inferiority trial that compares a novel combined protocol for treating uncomplicated acute malnutrition compared to the current standard protocol in South Sudan and Kenya. We will calculate the total economic costs of both protocols from a societal perspective, using accounting data, interviews and survey questionnaires. The incremental cost of implementing the combined protocol will be estimated, and all costs and outcomes will be presented as a cost-consequence analysis. Incremental cost-effectiveness ratio will be calculated for primary and secondary outcome, if statistically significant.

**Discussion:**

We hypothesise that implementing the combined protocol will be cost-effective due to streamlined logistics at clinic level, reduced length of treatment, especially for MAM, and reduced dosages of RUTF. The findings of this economic evaluation will be important for policymakers, especially given the hypothesised non-inferiority of the main health outcomes. The publication of this protocol aims to improve rigour of conduct and transparency of data collection and analysis. It is also intended to promote inclusion of economic evaluation in other nutrition intervention studies, especially for MAM, and improve comparability with other studies.

**Trial Registration:**

ISRCTN 30393230, date: 16/03/2017.

**Electronic supplementary material:**

The online version of this article (10.1186/s13063-018-2594-7) contains supplementary material, which is available to authorized users.

## Background

Tackling undernutrition in all its forms is a major global health priority demonstrated by its inclusion in the recent Sustainable Development Goals [1]. Stunting, severe acute malnutrition (SAM) and intrauterine growth restriction together are responsible for 21% of disability-adjusted life-years for children under 5 years [2]. Annually, acute malnutrition specifically affects 52 million children aged under 5 years and is associated with a high mortality rate if untreated as well as multiple long-term implications [3, 4]. Acute malnutrition is currently defined as low weight-for-height, low mid upper arm circumference (MUAC), and/or presence of bilateral oedema. It can be either moderate (MAM) or severe (SAM), depending on the extent of wasting. SAM cases, without medical complications, are treated through outpatient therapeutic programmes, where they receive routine medical care and a weekly take-home ration of ready-to-use therapeutic food (RUTF). Although there is currently no consensus on how best to manage MAM cases, they are often treated in food-insecure settings through supplementary feeding programmes (SFP), where they receive bi-weekly take-home rations of ready-to-use supplementary food [5, 6]. When discussing SAM and MAM in combination, the term ‘global acute malnutrition’ (GAM) is applied.

The current system of two parallel malnutrition treatment programmes and product supply chains means that SAM programmes are often prioritised over those for MAM, resulting in no services for MAM children in many settings. In contexts where the treatment of both SAM and MAM are available, the parallel systems may be resulting in an inefficient use of resources. In addition, current dosage of RUTF for treatment of SAM is based on the weight of the child, requiring multiple calculations by health workers and, in some cases, children are provided with a higher dose and for a longer period of time than required [7].

ComPAS (Combined Protocol for Acute Malnutrition Study) aims to assess whether unifying and simplifying the treatment of uncomplicated SAM and MAM for children aged 6–59 months into one programme would have an impact on the effectiveness of treatment. The ‘combined protocol’ will treat all SAM and MAM cases with RUTF using a simple MUAC-based dosage protocol, and this will be compared to the ‘standard protocol’ in a non-inferiority, cluster-randomised controlled trial. Further details of the ComPAS trial study design are published elsewhere [7, 8].

Few studies have assessed the cost-effectiveness of nutrition interventions, particularly with regard to treatment of MAM and changes in acute malnutrition protocols. Since the introduction of the current standard community-based protocol for treatment of SAM in 2001, studies have found this model to be cost-effective compared to the previous model of inpatient treatment [9–11]. Treatment for SAM is estimated to cost between USD$26 and USD$53 per disability-adjusted life year averted [9–11], and between USD$100 and USD$203 per child treated [11, 12]. It has also been estimated that costs can be as high as USD$500 per child treated by non-government organisations in fragile or emergency contexts [13]. The cost per MAM child treated is not widely published, perhaps due to the current lack of recommended protocols.

One previous study has trialled a combined protocol, using RUTF to treat MAM and SAM, and found it to have a comparable recovery rate (83% vs. 79%) for GAM and higher coverage (71% vs. 55%, *P* = 0.0005) than the standard protocol [14]. This clinical trial was conducted in Sierra Leone in 2013 and tested the efficacy of an integrated, MUAC-only protocol for the treatment of SAM and MAM using one product (RUTF) at different standardised doses for children with a height < 115 mm (175 kcal/kg/day) and 115–125 mm (75 kcal/kg/day), against the standard protocol, which used corn-soy blended flour (CSB++) for children with MAM. The study also presents the cost of therapeutic food from a programme perspective. The cost of RUTF was US$4/kg, which was the local producer’s price in 2013, and US$1.30/kg for CSB++ [14]. The cost of RUTF used to treat a SAM case was US$36 in the combined protocol, compared to US$68 in the standard programme. The cost of food products to treat a case of MAM was US$12 in both the combined and standard management arms.

Despite the higher price of RUTF compared to CSB++, the study found that the integrated protocol was less costly due to the lower dose of RUTF provided to SAM cases and faster recovery rates of children with MAM. The authors theorised that, due to the reduced food costs and simpler logistical requirements, it seemed likely that the integrated management would be less costly to implement per child treated than the standard protocol [14]. They also found a lower proportion of SAM in their combined protocol arm, which they hypothesised might be due to SAM cases averted by earlier treatment of MAM with RUTF. The need for a more complete economic analysis of the combined protocol was highlighted.

**Fig. 1:**
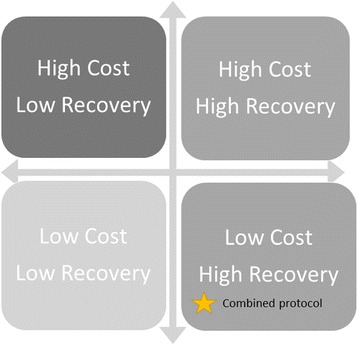
Depiction of hypothesised economic outcome of the ‘combined protocol’ on the cost-effectiveness matrix. We hypothesise that the ‘combined protocol’ for treatment of severe and moderate acute malnutrition will have as high recovery rates as the standard treatment protocol, but at a lower cost

Our study builds on this previous work, and aims to estimate the full economic cost of the ComPAS combined protocol, compared to the standard protocol, from a societal perspective. We hypothesise that the combined protocol will be more cost-effective than the standard protocol as overall costs will be lower and an equivalent recovery rate from GAM will be achieved. Many cost-effective health interventions have better health outcomes but at a greater cost; we hypothesise that the combined protocol will have as high recovery rates as the standard protocol, at a lower cost (Fig. 1). Based on the literature, we hypothesise that cost reductions will come from the streamlining of logistics and personnel, the potential for provision of lower weekly dosages of RUTF, and the faster recovery of children.

As few protocols have been published for economic evaluations in child health, this article follows a call for publication of complex economic protocols [15]. The publication of economic protocols, such as this, aims to improve the rigour of conduct among costing studies, improve comparability, and familiarise researchers and programme implementers in global health nutrition with the methodologies of economic evaluation in order to encourage its inclusion in more nutrition intervention trials.

### Aims and objectives

The aim of the ComPAS economic evaluation is to measure the cost and cost-effectiveness of a ‘combined protocol’ for treatment of SAM and MAM compared to the standard protocol, which separates SAM and MAM treatment into an outpatient therapeutic programme and SFP. The primary marker of effectiveness will be recovery from GAM, defined as achieving a MUAC ≥ 125 mm. The secondary markers of effectiveness will be programme coverage, length of treatment, treatment adherence, average weekly weight gain and average weekly MUAC gain.

The specific objectives of the economic evaluation are to:

1. Quantify the economic cost of implementing the combined protocol (intervention) and the standard protocol (control), using a combination of activity-based costing and ingredients approach.
2. Quantify the economic cost to households partaking in the combined protocol and the standard protocol.
3. Estimate any incremental costs to the wider health system by the implementation of the combined protocol compared to the standard protocol using programme referral data and published literature.
4. Compute the incremental cost per child recovered for the combined protocol compared to the standard protocol.
5. Present the costs and any significant differences in the primary and secondary outcomes (i.e. recovery rate, coverage, defaulting, average weight gain, average MUAC gain and length of treatment) as a cost-consequence analysis.

## Methods

### Study design

This study is a cost-consequence and cost-effectiveness analysis of the ComPAS trial, a multi-country, cluster-randomised, controlled trial taking place between May 2017 and July 2018. We will estimate the total costs of both protocols from a societal perspective, including both service provider and household costs. Data collection for the costing study will take place at the mid-point of recruitment for the main trial, and accounting data will be accessed at the end of the study implementation. Time horizons for the costing study are within the trial period only.

Ethical approval for the trial was obtained from the Kenya Medical Research Institute (KEMRI) (5/1/2017, ref.: 551), the South Sudan Ministry of Health Internal Review Board (21/11/2016), and the London School of Hygiene and Tropical Medicine (28/11/2016, ref: 11826). This protocol describes the methods specific to the economic study alongside the ComPAS randomised controlled trial, following the SPIRIT checklist format (see Additional file 1); further details of the protocol have been published elsewhere [8].

### Target population

The target population for the ComPAS trial is children aged 6–59 months who are diagnosed with uncomplicated acute malnutrition and are eligible for outpatient treatment. Acute malnutrition is defined in the trial as a MUAC < 125 mm and/or bilateral pitting oedema only. Children with a weight-for-height < −2 z-score will be treated, however, they will not be included in the primary analysis. The only reason for non-inclusion will be if the child is receiving SAM or MAM treatment at another facility.

All caregivers of children presenting with acute malnutrition at participating health facilities, who meet the inclusion criteria, are approached for consent to take part in the trial. Details of the informed consent process can be found in the trial protocol [8]. If the caretaker chooses not to participate in the trial, their child is enrolled for treatment only and their information is not recorded for study purposes.

For the economic evaluation, the target populations for the collection of resource-use data are (1) carers of children receiving treatment for acute malnutrition as part of the trial; (2) staff working to treat children enrolled in the trial; (3) support, supervision, management and logistics staff relevant to the treatment of children in the trial; and (4) partner organisations supporting any aspect of care provision to children enrolled in the trial.

### Setting and randomisation

The study will take place in 12 health facilities in Nairobi County in Kenya and 12 health facilities in Aweil East County, South Sudan. Six health facilities in each country have been randomised to the control arm and six to the intervention arm using a random sequence generated and applied to a pre-written list of participating clinics. Health facility staff are not blinded to the trial, as they are required to follow the specific protocol to which their facility is allocated.

Nairobi County is an urban area where more than 60% of residents live in informal settlements, in which the estimated prevalence of wasting (GAM) is 2.8% [16]. The 2014 Kenya Demographic and Health Survey estimated that 17% of children aged under 5 years in Nairobi were stunted, and 4% were underweight; further 60.4% of children were fully immunised and 93.6% of women living in urban areas were literate [17]. The 12 health facilities in Nairobi are operated by the Ministry of Health with support from International Rescue Committee specifically for treatment of malnutrition. These facilities were selected as study sites by the Ministry of Health based on the high burden of malnutrition; they are approximately 3–5 km apart.

Aweil East is a rural setting in Northern Bahr el Ghazal State in South Sudan. Children under 5 years of age are thought to account for 19% of the population and 76% of the population in the State live below the poverty line [18]. A Standardized Monitoring and Assessment of Relief and Transitions survey conducted in Aweil East in April 2014 reported a GAM prevalence of 26% [19]. Coverage of measles immunisations is thought to be 27%, and the 2009 National Baseline Household Survey found national female literacy rates to be 16% [20]. The 12 health facilities included in this study are operated by an international non-government organisation (Action Against Hunger), are approximately 20–30 km apart from each other and provide malnutrition services only.

### Intervention

The control arm follows the standard protocol defined by national guidelines for the treatment of SAM and MAM, and the intervention arm follows a combined protocol for SAM and MAM. The RUTF dosage for the intervention was developed based on a retrospective analysis of acute malnutrition treatment data in order to propose a physiologically appropriate, simplified dosage that meets the energy requirements for acutely malnourished children defined by MUAC [7]. Children treated in the control arm receive 200 kcal/kg/day of RUTF if they are classified as SAM (MUAC < 115 mm or bilateral pitting oedema), and 500 kcal/day of ready-to-use supplementary food if they are classified as MAM (MUAC 115 to < 125 mm), as per national protocols. Children treated in the intervention arm receive 1000 kcal/day of RUTF if they have SAM and 500 kcal/day of RUTF in they have MAM. Further details of the two protocols can be found in Table two of Bailey et al. [8] and in the online trial registration (ISRCTN 30393230) [21].

### Health outcomes

The primary outcome of the ComPAS trial is ‘proportion of children recovered’; however, as a non-inferiority trial, it is expected that this outcome will not be statistically significantly different between intervention and control arms. For the purposes of analysis, recovery will be defined as two consecutive measurements with a MUAC ≥ 125 mm and no oedema for both control and intervention arms.

The secondary outcomes will be treatment coverage, defaulter rate, length of treatment, average weight gain and average MUAC gain. Coverage is defined as proportion of children eligible for treatment (MUAC < 125 mm) who receive treatment, assessed by a standardised Semi-Quantitative Evaluation of Access and Coverage survey [22]. Defaulting is defined as absence from treatment appointments for 3 consecutive weeks. Further details of health outcomes can be found in the trial protocol [8]. Statistically significant secondary outcomes will be presented in the cost-consequence analysis and unit costs presented where appropriate, e.g. cost per unit coverage, although the conservative *a priori* hypothesis for all outcomes is non-inferiority.

Maust et al. [14] found a reduced caseload of SAM in their version of the combined protocol and hypothesised that this could be due to more intensive treatment of MAM resulting in prevention of SAM. We will also explore any adjusted differences in SAM caseloads between the intervention and control arm and, if a significant difference is found, the cost per SAM case averted will be estimated. They also found that SAM children who received the intervention recovered more rapidly and therefore had a reduced cost of RUTF [14]. We will also present the cost of RUTF per SAM case simply using the number of sachets prescribed and the local unit cost per sachet, in order to compare with previously published values. Nevertheless, it is important to note that the study by Maust et al. [14] compared their combined protocol to routine control clinics, whereas this study will compare a combined protocol to fully supported, ‘optimal’ control sites. Exploration of differences in defaulting rates and relapse rates between the two protocols will also be considered in relation to their effect on total programme costs.

Recent health seeking and referrals to external medical care will also be assessed in a sub-set of study participants in order to estimate differences in health seeking behaviour and utilisation of wider health services, which will inform the estimated cost of the intervention to the wider health system.

### Sample size

There will be 12 health facilities (clusters) in South Sudan and 12 in Kenya; in each country, six health facilities will be randomly allocated as intervention and six as control, with 150 children per clinic. The sample size for the main trial outcome was calculated using an expected recovery rate of 85% based on the average programme statistics for each site, allowing for a 10% non-inferiority margin, with 80% power at the 5% level of significance.

As the study is only statistically powered for determining effectiveness across both countries rather than within each country, the economic evaluation will be conducted using pooled costs and outcomes from both sites. Pooled and site-specific costs and outcomes will also be presented in the cost-consequence analysis.

Cost data collection will be carried out at all 24 health facilities participating in the study. For beneficiary costs, an exhaustive sample will be asked a simple survey about cost and time implications of participating in the programme as part of the main trial exit interview. In addition, more in-depth group interviews with beneficiary carers will take place with between four and six purposively selected participants at each clinic (i.e. ~72 participants for each country) (Table 1). Interview sample sizes will be determined by data saturation. Survey sample sizes will be dictated by the trial sample size; however, post-hoc sample size calculations for differences in cost and time data will be calculated where possible.

**Table 1:** Outline of data categories and sources

Activity	Ingredients	Data sources	Sample size
Programme costs			
Treatment^a^	Therapeutic food	1. MoH/partner accounting data 2. Clinic audit 3. KII with partners (UN agencies) 4. Time allocation questionnaire	1. N/A 2. All clinics (12 per country) 3. One from each partner 4. Purposive sample representing all types of clinic staff involved in treatment
	Human resources		
	Other medical supplies		
	Other supplies		
	Space		
Outreach	Transport	1. MoH/partner accounting data 2. Clinic audit	1. N/A 2. All clinics (12 per country)
	Supplies		
	Human resources		
Supply logistics	Storage	1. MoH/partner accounting data 2. KII with partners and clinic staff 3. Time allocation questionnaire	1. N/A 2. Purposive sample representing all partners 3. Purposive sample representing all types of clinic staff
	Transport		
	Human resources		
Training	Space	1. MoH/partner accounting data 2. Clinic audit	1. N/A 2. All clinics (12 per country)
	Supplies		
	Human resources		
Supervision	Transport	1. KII with supervisors	1. Purposive sample representing all supervisors
	Human resources		
Management	Human resources	1. MoH/partner accounting data 2. KII with MoH/partner support staff 3. Time allocation questionnaire	1. N/A 2. Purposive sample 3. All support staff involved in the programme
	Equipment		
	Space		
Household costs			
Participating in treatment	Supplies	1. GIs with beneficiaries 2. Beneficiary exit survey	1. 4–6 randomly selected beneficiaries from each clinic 2. All beneficiaries
	Time		
	Transport		
Wider health system costs			
Referral to other healthcare providers	Space	1. Clinic audit 2. Literature estimates	1. All clinics (12 per country) 2. N/A
	Human resources		
	Supplies		

^a^Note that this does not include SAM inpatient treatment

*KII* key informant interview, *MoH* Ministry of Health, *GIs* group interviews, *N/A* not applicable

### Measuring resources and costs

**Fig. 2:**
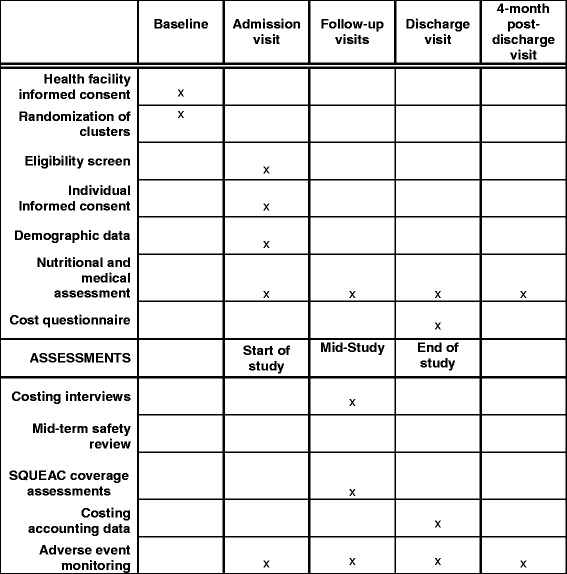
Schedule of enrolment, interventions and assessments

The economic evaluation will be conducted from a societal perspective, measuring programme and household costs. A combination of activity-based costing and ingredients approach will be used to estimate programme costs. See Table 1 for list of activities and ingredients. The timing of data collection points can be seen in Fig. 2. Full start-up costs will not be collected as protocol development has been on-going across previous years and previous studies; however, simple start-up costs relevant for future use, such as stakeholder meetings, trainings, community sensitisation and translation of resources, will be estimated. Note that cost of inpatient treatment for SAM will not be collected as it is not part of the intervention.

#### Measuring programme costs

Major activities guiding the programme costs have been derived from other community management of acute malnutrition costing analyses [9, 23], namely treatment, outreach, supply logistics, training, supervision and management. The ingredients within each activity will be quantified and costed using a clinic audit, key informant interviews with clinic staff, Ministry of Health regional level staff and relevant partner organisations, and accounting data, where available (Table 1). Capital costs will be annualised over their expected useful life (3 years for computers, 5 years for cars and other equipment, 10 years for communal land) and discounted at a rate of 3%. The allocation of joint costs will be divided by activity and by study arm, informed by the weekly clinic survey and study-midpoint staff time-use interview.

As programme costs are the primary area where we anticipate the main differences to lie between intervention and control, we will collect primary resource use data for all activity areas rather than rely on assumptions applied to accounting data. Specifically, we hypothesise differences in therapeutic food and staff costs.

#### Measuring household costs

Basic travel time and costs will be measured using survey questions to all study participants during the main trial exit interview. In Kenya, survey data will be collected electronically and stored on a secure server. In South Sudan, survey data will be collected on paper and entered using the same electronic data collection system as in Kenya, which includes appropriate skip logic, value restrictions and data checks. Group interviews with a sub-sample of participants during their enrolment in the study will also be undertaken to consider any hidden or abstract costs to households as well as to calculate potential lost earnings due to time spent at the programme. Participants will be purposively selected to represent both SAM and MAM children as well as a range of travel times.

#### Estimating costs to the wider health system

Wider health system costs will be estimated using the number and nature of referrals of participating children as well as any differences in health-seeking behaviour. Referral nature and number will be estimated based on quarterly clinic surveys and health-seeking behaviour will be discussed in participant group interviews. Cost will be estimated using existing published data on costs of treating common referrals, for instance WHO CHOICE cost estimates [24].

### Analytical methods

Incremental cost-effectiveness ratios will be calculated for the primary outcome (i.e. incremental cost per additional child recovered) and for secondary outcomes where a statistically significant difference is found. Potential secondary outcomes for inclusion are treatment coverage, defaulter rate, length of treatment, average weight gain and average MUAC gain. Univariate and multivariate sensitivity analyses will be performed in order to assess the potential best case and worse case variability in costs and outcomes. Areas of potential variability will be determined through exploration of the effectiveness data and the researcher’s evaluation of cost data uncertainty following data collection. For example, there could be variability in costs or time-use information provided by staff and other key informants due to recall error.

In addition to incremental cost-effectiveness ratios, the economic evaluation will be presented as a cost-consequence analysis where all costs and outcomes will be listed, including the multiple secondary trial outcomes, allowing policymakers to compare costs with health gains for the combined protocol.

Costs will be presented in 2017 prices in local currency (Kenyan shillings and South Sudanese Pounds) as well as 2017 US dollars, which will allow for comparison across the two country sites as well as comparison with other studies. Costs and outcomes will not be discounted as the intervention does not span more than 1 year.

Results are expected to be generalisable to multiple African contexts, particularly within the range of the sensitivity analyses. Household food security estimates for each site will aid identification of where results may be generalisable to (i.e. food insecure settings). In addition, as treatment in one study site is government-led and in the other it is partner-led, results from each should be of interest to implementers with a variety of programme structures. One limitation of the study is that the main analysis will present an average of the two sites, therefore masking some of the heterogeneity between sites.

Anonymised hardcopies of data will be stored in approved, secured locations in the respective countries; anonymised electronic data for the economic evaluation will be stored on a secure server in the UK, assessable only by the research team. No participant identifiable data will be recorded. Results will be reported following the consolidated health economic evaluation reporting standards (CHEERS) checklist [25].

## Discussion

This economic evaluation will provide key information for policymakers when considering alterations to the GAM treatment protocols. As a non-inferiority trial, the recovery rates are expected to be statistically similar between each protocol, hence the comparative cost-effectiveness will likely play a larger role in policy decision-making.

With regard to limitations, this study will not be able to assess the cost impact of the combined protocol on international logistics and supply chain, which may be further streamlined if the combined protocol were to be scaled-up. In addition, the full extent of the hypothesised cost savings of the combined protocol is unlikely to be realised during the period of this study (8–10 months). A longer time horizon is necessary to reveal the impact of an improved treatment protocol on the reduction in acute malnutrition caseload through prevention of deterioration from MAM to SAM. Finally, as is the case with many controlled trials, the extra support offered to both the combined and standard protocols will likely result in higher costs, which should be noted when generalising the results to routine programmes.

This publication aims to improve rigour in conduct and transparency of data collection and analysis. The publication of this protocol also intends to promote inclusion of economic evaluation in other nutrition intervention studies, particularly for MAM, and improve comparability with other studies. Regardless of whether the new protocol is more cost-effective than the standard protocol or not, the evidence provided by this evaluation will contribute significantly to the currently limited evidence regarding cost-effectiveness of nutrition-specific interventions.

## Trial status

This is protocol version number 1, finalised on 01/06/2017. Recruitment of trial participants began on 15/05/2017 and is expected to be completed on 01/06/2018, at which point accounting data and survey data will be analysed. Interviews for the economic evaluation began on 20/06/2017 and will be completed on 20/12/2018.

## Additional files

Additional file 1: SPIRIT 2013 Checklist: Recommended items to address in a clinical trial protocol and related documents. (DOC 132 kb)

Additional file 2: Caretaker consent form for group interviews (Kenya). (DOCX 26 kb)

## Abbreviations

ComPAS: Combined Protocol for Acute Malnutrition Study
CSB++: corn-soy blend (fortified)
GAM: global acute malnutrition
MAM: moderate acute malnutrition
MUAC: mid upper arm circumference
RUTF: ready to use therapeutic food
SAM: severe acute malnutrition
SFP: supplementary feeding programme
