# Supplementary material for: The "ComPAS Trial" combined treatment model for acute malnutrition: study protocol for the economic evaluation
Source: Trials. 2018 Apr 24;19:252. doi: 10.1186/s13063-018-2594-7 (PMC5916722; doi:10.1186/s13063-018-2594-7)
Supplement: Supplementary file 2 — Caretaker consent form for group interviews (Kenya). (DOCX 26 kb) [file 13063_2018_2594_MOESM2_ESM.docx]

# Additional file 2: Caretaker Consent for Group Interviews (Kenya)

**INFORMED CONSENT DOCUMENT**

**Recruitment Consent Form (Caretakers-FGD)**

Study Title: **Com**bined **P**rotocol for **A**cute **M**alnutrition **S**tudy (ComPAS)

Study Coordinator: Bethany Marron, +254 (0) 780 419 011

You are being asked to voluntarily participate in an interview that is being conducted by International Rescue Committee, the Ministry of Health, and London School of Hygiene and Tropical Medicine because your child has been receiving a food ration from the OTP or SFP at the Community Health Center. In a group setting, we would like to get your views on the time and cost of participating in the program. The whole discussion will take about 1 hour. The reason for the discussion is to understand what the program costs for everyone in order to inform other programs.

If you decide to take part in this discussion, then we will do the following:

1. We will ask you to come to wait at the Community Health Center or come at a specific time to join other caretakers for this discussion.
2. In a group of 6 other caretakers, we will ask you about your travel time, your expenses and your normal job.
3. We may take some pictures during the focus group discussion. These pictures will not identify you by name. You can still participate in the discussion if you do not agree to have your picture taken.
4. We will be recording what you say during the group discussion. This will not identify you by name. You can still participate in the discussion if you do not consent to have your comments recorded. See below.

Participation in this interview is voluntary. If you choose to not participate in the interview, your child will still continue receiving the same food. We do not think there will be any risks from participating in this interview. We will do everything to protect your privacy. We will be happy to answer any questions you have about this study. If you have a project worry, you may contact the Research Coordinator, Bethany Marron XXXXXX. If you have question about your rights as a research study subject, call the Kenya Medical Research Institute Institutional Review Board (IRB) at XXXXXX. The IRB is a group of doctors and non-medical people who review human research studies for safety and protection of people who take part in the studies.

Although there is no direct benefit to participation, the study will benefit other caretakers and children in Kenya and elsewhere by helping us understand how to improve their nutrition and health. The discussion will be audio-recorded, but the recordings will only be used to transfer the information into a computer. The records of this study will be kept securely, in locked cabinets in the study coordinator’s office. Your information will not be shared with anyone outside of the research team. The results of the study will not be connected to you as an individual or to your responses. You can still participate in the discussion if you do not consent to be audio-recorded, the device will be turned off while you speak. Please understand the risks of other participants possibly sharing the information shared in these groups.

If you take part in this study, it will not cost you or any member of your family any money. You will have to find your own way of coming to the meeting point. The study team will reimburse you $3 for travel costs. During the FGD we will provide you with light refreshments.

*A description of this clinical trial will be available on http://www.Clinical Trials.gov, as required by US Law. This Web site will not include information that can identify you. At most, the Web site will include a summary of the results. You can search this web site at any time.*

**Caretaker’s Consent for her Participation**

YOUR SIGNATURE OR THUMBPRINT, BELOW, WILL SHOW THAT YOU HAVE DECIDED TO VOLUNTEER AS A RESEARCH PARTICIPANT AND THAT YOU HAVE READ OR RECEIVED CLEAR DESCRIPTION OF THE STUDY, AND UNDERSTOOD THE INFORMATION PROVIDED ABOVE, AND YOUR RIGHTS AS A PARTICIPANT.

Participant Signature/Thumbprint _________________ Name_______________________________

Interviewer Signature/ Thumbprint _________________ Name_______________________________

**Guardian Consent for a Minor**

INSTRUCTIONS FOR ENUMERATOR:

a. If mother/caretaker is under 18yrs and stays with an elderly person/guardian, guardian must provide consent

b. If mother/caretaker is under 18 yrs and stays with husband or man responsible for the child, the man must provide consent

c. If mother/caretaker is under 18 yrs but is independently living on her own. She can give consent herself]

Guardian Signature/Thumbprint _________________ Name_____________________

Interviewer Signature/Thumbprint __________________ Name _____________________

IF A GUARDIAN’S SIGNATURE IS REQUIRED, THEY WILL CONSENT FOR THE CARETAKER ON ALL OTHER AREAS OF THIS FORM.

**PHOTOGRAPHS**

There may be photos taken during the interview. These will not identify you by name. You can still be interviewed if you choose to not have your photo taken.

**Caretaker’s consent for photographs and audio recording**

PUTTING YOUR SIGNATURE OR THUMBPRINT BELOW WILL SHOW THAT YOU AGREE TO BEING PHOTOGRAPHED and audiotaped DURING THE INTERVIEW.

Participant or Guardian
Signature/Thumbprint _________________ Name_______________________________

# Caretaker Consent for Focus Group Discussion (South Sudan)

**INFORMED CONSENT DOCUMENT**

**Recruitment Consent Form (Caretakers-FGD)**

Study Title: **Com**bined **P**rotocol for **A**cute **M**alnutrition **S**tudy (ComPAS)

Study Coordinator: Pamela Onyoo, Phone Number: XXXXX

You are being asked to voluntarily participate in an interview that is being conducted by Action Against Hunger, the Ministry of Health, and London School of Hygiene and Tropical Medicine because your child has been receiving a food ration from the OTP or SFP. In a group setting, we would like to get your views on the time and cost of participating in the program. The whole discussion will take about 1 hours. The reason for the discussion is to understand what the program costs for everyone in order to inform other programs.

If you decide to take part in this discussion, then we will do the following:

1. We will ask you to come to wait at the OTP/SFP or come at a specific time to join other caretakers for this discussion.
2. In a group of 6 other caretakers, we will ask you about your travel time, your expenses and your normal job.
3. We may take some pictures during the focus group discussion. These pictures will not identify you by name. You can still participate in the discussion if you do not agree to have your picture taken.
4. We will be recording what you say during the group discussion. This will not identify you by name. You can still participate in the discussion if you do not consent to have your comments recorded. See below.

Participation in this interview is voluntary. If you choose to not participate in the interview, your child will still continue receiving the same food. We do not think there will be any risks from participating in this interview. We will do everything to protect your privacy. We will be happy to answer any questions you have about this study. If you have a project worry, you may contact the Research Coordinator, Pamela Onyoo XXXXX. If you have question about your rights as a research study subject, call the South Sudan Ministry of Health Review Board (IRB) at: +211 (0) 177800281. The IRB is a group of doctors and non-medical people who review human research studies for safety and protection of people who take part in the studies.

Although there is no direct benefit to participation, the study will benefit other caretakers and children in Kenya and elsewhere by helping us understand how to improve their nutrition and health. The discussion may be audio-recorded, but the recordings will only be used to transfer the information into a computer. The records of this study will be kept securely, in locked cabinets in the study coordinator’s office. Your information will not be shared with anyone outside of the research team. The results of the study will not be connected to you as an individual or to your responses. You can still participate in the discussion if you do not consent to be audio-recorded, the device will be turned off while you speak. Please understand the risks of other participants possibly sharing the information shared in these groups.

If you take part in this study, it will not cost you or any member of your family any money. You will have to find your own way of coming to the meeting point. The study team will reimburse you $2 for travel costs. During the FGD we will provide you with light refreshments.

*A description of this clinical trial will be available on http://www.Clinical Trials.gov, as required by US Law. This Web site will not include information that can identify you. At most, the Web site will include a summary of the results. You can search this web site at any time.*

**Caretaker’s Consent for her Participation**

YOUR SIGNATURE OR THUMBPRINT, BELOW, WILL SHOW THAT YOU HAVE DECIDED TO VOLUNTEER AS A RESEARCH PARTICIPANT AND THAT YOU HAVE READ OR RECEIVED CLEAR DESCRIPTION OF THE STUDY, AND UNDERSTOOD THE INFORMATION PROVIDED ABOVE, AND YOUR RIGHTS AS A PARTICIPANT.

Participant Signature/Thumbprint _________________ Name_______________________________

Interviewer Signature/ Thumbprint _________________ Name_______________________________

**Guardian Consent for a Minor**

INSTRUCTIONS FOR ENUMERATOR:

a. If mother/caretaker is under 18yrs and stays with an elderly person/guardian, guardian must provide consent

b. If mother/caretaker is under 18 yrs and stays with husband or man responsible for the child, the man must provide consent

c. If mother/caretaker is under 18 yrs but is independently living on her own. She can give consent herself]

Guardian Signature/Thumbprint _________________ Name_____________________

Interviewer Signature/Thumbprint __________________ Name _____________________

IF A GUARDIAN’S SIGNATURE IS REQUIRED, THEY WILL CONSENT FOR THE CARETAKER ON ALL OTHER AREAS OF THIS FORM.

**PHOTOGRAPHS**

There may be photos taken during the interview. These will not identify you by name. You can still be interviewed if you choose to not have your photo taken.

**Caretaker’s consent for photographs and audio recording**

PUTTING YOUR SIGNATURE OR THUMBPRINT BELOW WILL SHOW THAT YOU AGREE TO BEING PHOTOGRAPHED and audiotaped DURING THE INTERVIEW.

Participant or Guardian
Signature/Thumbprint _________________ Name_______________________________
